# The Impact of Psychological Capital on Job Burnout of Chinese Nurses: The Mediator Role of Organizational Commitment

**DOI:** 10.1371/journal.pone.0084193

**Published:** 2013-12-27

**Authors:** Jiaxi Peng, Xihua Jiang, Jiaxi Zhang, Runxuan Xiao, Yunyun Song, Xi Feng, Yan Zhang, Danmin Miao

**Affiliations:** Department of Psychology, Fourth Military Medical University, People's Republic of China; University of Leicester, United Kingdom

## Abstract

**Background:**

Nursing has a high risk of job burnout, but only a few studies have explored its influencing factors from an organizational perspective.

**Objective:**

The present study explores the impact of psychological capital on job burnout by investigating the mediating effect of organizational commitment on this relationship.

**Methods:**

A total of 473 female nurses from four large general hospitals in Xi’an City of China were selected as participants. Data were collected via the Psychological Capital Questionnaire, the Maslach Burnout Inventory-General Survey, and the Organizational Commitment Scale.

**Results:**

Both psychological capital and organizational commitment were significantly correlated to job burnout. Structural equation modelling indicated that organizational commitment partially mediated the relationship between psychological capital and job burnout.

**Conclusion:**

The final model revealed a significant path from psychological capital to job burnout through organizational commitment. These findings extended prior reports and shed some light on the influence of psychological capital on job burnout.

## Introduction

Occupational happiness reflects the subjective well-being of individuals at the workplace, and refers to the positive and negative emotional feelings of employees towards their jobs as well as their cognitive evaluations of their jobs[1-3]. On the one hand, researchers use indicators, such as job satisfaction, work engagement, positive emotional experience at the workplace, as indirect measures of employee well-being[4-7]. On the other hand, researchers are paying increasing attention to other indicators, such as turnover intentions, job strain, job burnout, as negative predictors of employee well-being at the workplace[8,9].

Job burnout is a state of mental and physical exhaustion that results from prolonged heavy workload and stress. In 1974, American psychiatrist Freudenberger first proposed the concept of “job burnout” to describe unhealthy physical, psychological, and emotional states, such as fatigue, decreased work engagement, reduced sense of accomplishment, which were experienced by people working in human service professions due to long working hours, heavy workload, and excessive work intensity[10]. Maslach and Jackson further defined “job burnout” as long-term stress response of an individual to prolonged exposure to emotional and interpersonal stressors at work, which encompass emotional exhaustion, depersonalization, and reduced personal accomplishment[11]. Emotional exhaustion refers to feelings of extreme emotional fatigue and lack of enthusiasm and vigor towards work. Depersonalization refers to the deliberate attempt to maintain distance between the self and work as well as the exhibition of passive, indifferent, and cynical attitudes and emotions towards others at work. Reduced personal accomplishment is manifested in low sense of self-respect and in even more negative evaluation of work, inability to experience pleasure, satisfaction, and a sense of accomplishment associated with performing the job. From a compositional perspective, job burnout comprises emotional experiences and cognitive components. If an individual is not passionate for his or her job, has poor interpersonal relationships at work, and cannot find a sense of self-worth from performing his or her job, then the person will not experience happiness in his or her work. Therefore, we think that job burnout can serve as a strong negative measure of employee occupational happiness, and enhancing employee occupational happiness requires the reduction of job burnout. How can we reduce job burnout? This study focuses on both individual and organizational dimensions, namely, psychological capital and organizational commitment, in reducing job burnout.

In 2004, Luthans et al. proposed the concept of psychological capital within the framework of positive psychology and positive organizational behavior[12,13]. They defined psychological capital as “a positive state of mind exhibited during the growth and development of an individual” that includes four core components of self-efficacy, optimism, resiliency, and hope. Self-efficacy refers to the confidence in being able to execute a task, ability to face challenges, and the will to succeed. An optimistic individual makes attributions for positive events and maintains a positive attitude towards the present and future. Resiliency refers to the ability to recover quickly, or even change and grow, from adversities, setbacks, and failures. Hope is the positive motivational state that helps in achieving the intended goals through various means. 

Several studies in management have shown that psychological capital and its various dimensions can have positive effects on work performance and attitudes of both leaders and employees. For instance, Avey, Patera, and West showed that each of the four dimensions of psychological capital, i.e., self-efficacy, optimism, resiliency, and hope, is negatively correlated with employee absenteeism and turnover intentions[14]. Luthans et al. conducted an empirical study of the relationship between psychological capital and job performance in 422 Chinese employees and found a positive correlation between the three positive mental states of hope, optimism, and resiliency, and the evaluation of work performance of these Chinese employees by their managers. Moreover, the positive correlation between psychological capital and work performance was even stronger, and psychological capital and employee performance-based pay were also positively correlated[15]. Currently, few studies have explored the relationship between psychological capital and job burnout. Luthans et al. believed that psychological capital plays a pivotal role in the development of job burnout and that it may effectively reduce the extent of burnout[13]. Using Chinese nurses as participants, Luo and Hao found preliminary evidence for the preventative effect of psychological capital on job burnout[16]. In general, however, the body of research concerning the relationship between psychological capital and job burnout remains relatively small. 

Organizational commitment refers to the attitude towards the organization. This attitude is a psychological bond in the relationship between an employee and the organization that affects the degree to which the individual identifies with the goals and values of the organization, exerts effort to achieve organizational goals, and desires to remain in the organization[17-19]. The three-factor model of organizational commitment proposed by Meyer and Allen, which includes affective, continuance, and normative[18], has been well-received and supported by international studies[20-22]. Affective commitment refers to the emotional attachment that employees feel towards their organization. Continuance commitment refers to the awareness of employees of the cost of losing their membership in the organization. Normative commitment refers to the level of consistency between the values of the individual and the organization or the sense of responsibility for the organization, which is shaped by the long-term influence of society on the sense of social responsibility of an individual. A large body of research supports the relationship between organizational commitment and job burnout. King and Sethi found that organizational commitment has a moderating effect on the relationship between stress and job burnout[23]. Tan and Akhtar reported that when age, tenure, organizational level, and work perceptions of Chinese employees were controlled, organizational commitment had a significant effect on experienced burnout[24]. Wright and Hobfoll further showed that organizational commitment has an effect on every dimension of job burnout[25]. A significant correlation also exists between psychological capital and organizational commitment. Luthans and Jensen demonstrated a very strong positive correlation between the psychological capital and the assessment of the commitment of nurses to the mission, values, and goals of the hospital[15]. In a study of 74 employees, Larson and Luthans reported a significant positive correlation between psychological capital and job satisfaction and organizational commitment[26]. Zhong studied the influence of psychological capital on work performance and organizational commitment in the Chinese cultural context and found, after controlling for gender and age, all three positive mental states of hope, optimism, and resiliency, had a positive influence on employee work performance, organizational commitment, and organizational citizenship; hence, psychological capital, which is composed of hope, optimism, and resiliency of an employee, had a positive impact on work performance, organizational commitment, and organizational citizenship[27].

Psychological capital can be seen as an important human resource that has significant effects on organizational commitment and job burnout. Organizational commitment can affect job commitment because an employee who has a sense of belonging and commitment to the organization is unlikely to tire from the job, and this effect can be even more significant in collectivist cultures[24]. While the relationship between any two of the three variables is relatively clear, the relationship of these three variables has not been explored. Nursing is a high risk, high pressure, and labor-intensive profession, and thus, a high incidence of job burnout exists among nurses. A survey conducted in five countries, including the United States, revealed that job burnout is a very serious phenomenon within the nursing profession. Among the five countries surveyed, 40% of the nurses in four countries experienced job burnout[28]. Nursing was also first in the job burnout profession ranking released in China in 2006. In the present study, we studied the relationship among psychological capital, organizational commitment, and job burnout in Chinese nurses.

## Methods

### 2.1: Participants and Procedure

The participants comprised 473 female nurses from four large general hospitals in Xi’an, China. Their ages ranged from 20 years to 39 years, with a mean of 26.23 years (SD = 3.60). At the time of the gathering of data, the nurses had worked in hospitals from 6 to 220 months. The participants completed the questionnaire in a classroom environment. All participants signed informed consent forms before completing the measures. The research described in this paper meets the ethical guidelines of the Fourth Military Medical University and was approved by the ethics committee of Xijing Hospital. Participants were told they were engaging in a psychological investigation in which there were no correct or incorrect answers. We distributed 473 questionnaires, which were all collected and validated. Participants received ¥40 in compensation.

### 2.2: Instruments

#### 2.2.1: Psychological Capital Questionnaire

The Psychological Capital Questionnaire (PCQ), developed by Luthans et al., is a 24-item self-report scale that includes four dimensions, namely, self-efficacy, optimism, resiliency, and hope[29]. The items are rated from 1 (strongly disagree) to 6 (strongly agree). Some of the items are “I usually take stressful things at work in stride” and “I always look on the bright side of things regarding my job.” Scale scores are the sum of items with reverse coding of relevant items. In this study, the Cronbach’s alpha coefficient for the PCQ was 0.846.

#### 2.2.2: Maslach Burnout Inventory-General Survey

The Maslach Burnout Inventory-General Survey (MBI-GS), developed by Maslach, is a 15-item self-report measure of job burnout that includes three dimensions, namely, emotional exhaustion, depersonalization, and reduced personal accomplishment[30]. The items are rated from 1 (never) to 7 (every day). Some items are “I have become less enthusiastic about my work,” and “I have become more cynical about whether my work contributes anything.” In this study, the Cronbach’s alpha coefficient for the MBI-GS was 0.884.

#### 2.2.3: Organizational Commitment Scale

The Organizational Commitment Scale (OCS), developed by Allen and Meyer, comprises 18 items and three dimensions, namely, affective, normative, and continuance[18]. The items are rated from 1 (strongly disagree) to 6 (strongly agree). Some items are “I am very happy being a member of this organization,” “I worry about the loss of investments I have made in this organization,” and “I feel that I owe this organization quite a bit because of what it has done for me.” Scale scores are the sum of items with reverse coding of relevant items. In this study, the Cronbach’s alpha coefficient for the OCS was 0.891.

### 2.3: Data Analysis

The mediation test is emphasized because we aimed to explore the trilateral relations among psychological capital, organizational commitment, and job burnout. Let X, M, and Y be the independent, mediating, and dependent variables, respectively, and let Y=cX+ e1, M=aX+e2, Y=c’X+bM+e3. The mediation effect is an indirect effect, in which the effect of independent variable on dependent variable goes through a mediator[31,32], which can be operationalized as a×b not equal to zero[33]. The commonly employed method for examining the statistical significance of a mediation effect is the Sobel test[34], which involves computing the ratio of a×b to its estimated standard error (Z= ab/$\sqrt{a^{2}{\text{s}}_{\text{b}}^{\text{2}}\text{+}b^{2}s_{a}^{2}}$). However, a Sobel test requires that a×b follows a normal distribution; otherwise, statistical efficacy would be reduced[35,36]. The bootstrap test implemented by Preacher and Hayes tested the null hypothesis a×b=0 in another way. This test takes sample size N and draws substitute N values of (X, M, Y) to create a new sample. If the option is repeated, for example, 1000 times, then 1000 estimations of a×b can be calculated[37]. The bootstrap test relies on 95% confidence intervals from the empirical distribution of a×b estimates[38].

In the current study, psychological capital, organizational commitment, and job burnout were regarded as latent variables, and thus, a two-step procedure introduced by Anderson and Gerbing was adapted to analyze the mediation effect[39]. First, the measurement model was tested to assess the extent to which each of the three latent variables was represented by its indicators. If the confirmatory measurement model were acceptable, then the maximum likelihood estimation would be used to test the structural model in the AMOS 17.0 program. The following four indices were used to evaluate the quality of the fit of the model: (a) Chi square statistic (χ^2^), (b) the Standardized Root Mean Square Residual (SRMR), (c) the Root Mean Square Error of Approximation (RMSEA), and (d) the Comparative Fit Index (CFI). A model was considered to have a good fit if all the path coefficients were significant at the level of 0.05, the SRMR was below 0.08, the RMSEA was below 0.08, and the CFI was 0.95 or higher[40,41].

Based on above, the bootstrap test and structural equation modelling were both used to test the mediation effect.

## Results

### 3.1: Measurement Model

Confirmatory factor analysis was used to examine whether the measurement model adequately fit the sample data. The measurement model included 3 latent constructs and 10 observed variables. The results indicated that the measurement model fit the data very well. For psychological capital: χ^2^ (239, N=473) =592.07, p<0.001; RMSEA=0.056; SRMR=0.076; and CFI=0.968. For organizational commitment: χ^2^ (128, N=473) =324.85, p<0.001; RMSEA=0.057; SRMR=0.048; and CFI = 0.951. For job burnout: χ^2^ (85, N=473) =258.72, p<0.001; RMSEA=0.056; SRMR=0.066; and CFI=0.967. All the factor loadings for the indicators on the latent variables were significant (P<0.001), indicating that the measurement model is acceptable. 

### 3. 2: Correlation Analyses

Table 1 shows all the latent variables, namely, psychological capital, organizational commitment, and job burnout, significantly correlated each other. 

**Table 1:** Inter-correlations between three latent variables.

	M	SD	1	2	3
1. psychological capital	97.5	15.41	1		
2. organizational commitment	60.36	9.59	0.47^**^	1	
3. job burnout	33.03	13.45	-0.58^**^	-0.37^**^	1

Note: ^*^, P<0.05, ^**^, P<0.01

### 3. 3: Structural Model and Bootstrap Test

**Figure 1:**
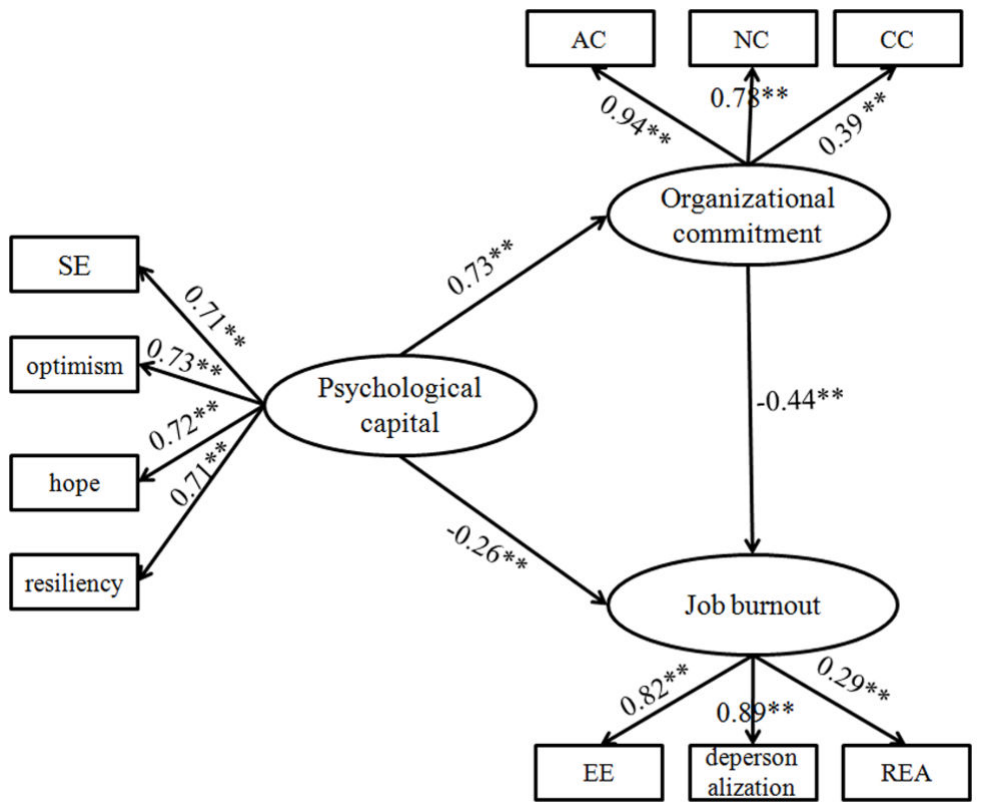
The final structural model (N = 310). Note: Factor loadings are standardized. *SE* self-efficacy, *EE* emotional exhaustion, *REA* reduced personal accomplishment, *AC* affective commitment, *CC* continuance commitment, *NC* normative commitment.

In the first step, the direct effect of the predictor (psychological capital) on the dependent variable (job burnout) in the absence of mediator (organizational commitment) was tested. The directly standardized path coefficient was significant, β=-0.60, p<0.001. After that, a partially mediated model (Model 1) that contained mediators (organizational commitment) and a direct path from psychological capital to job burnout was tested. The results showed that the model did not fit the data very well, χ^2^ (23, N=473) =190.02, p<0.001; RMSEA=0.084; SRMR=0.102; and CFI=0.921. However, an examination of parameter estimates indicated that the standardized path coefficient from psychological capital to job burnout and organizational commitment and from organizational commitment to job burnout were all significant. Thus, according to the modification indices in the Model 1, Model 2 was created by adding the correlations of residual terms between self-efficacy and optimism, between emotional exhaustion and reduced personal accomplishment, and between continuance commitment and normative commitment. After adding the correlations of the residual terms, the final meditational model, shown in Figure 1, was analyzed. The final meditational model showed a good fit to the data: χ^2^ (26, N=473) =94.68, p<0.001; RMSEA=0.054; SRMR=0.073; and CFI=0.966. Theoretically, the correlations of self-efficacy-optimism, emotional exhaustion-reduced personal accomplishment, and continuance commitment-normative commitment were easy to explain, and we deemed that the final model was acceptable. 

Lastly, the mediating effect of organizational commitment between psychological capital and job burnout was tested by adopting the bootstrap estimation procedure with AMOS (a bootstrap sample of 1,500 was specified). Table 2 shows psychological capital had significant direct effect on job burnout (β=-0.262, p<0.001). In addition, the indirect effect through organizational commitment was also significant (β=-0.321, p<0.001). The indirect effect made up 55.1% of the total. 

**Table 2:** Direct and indirect effects and 95 % confidence intervals for the final model.

Model pathways	Estimated effect	95% CI (Lower bonds)	95% CI (Up bonds)
**Direct effect**			
psychological capital→job burnout	-0.262**^*a*^**	-0.428	-0.106
psychological capital→organizational commitment	0.731**^*a*^**	0.652	0.799
organizational commitment→job burnout	-0.439**^*a*^**	-0.596	-0.273
**Indirect effect**			
psychological capital→organizational commitment→job burnout	-0.321**^*a*^**	-0.437	-0.201

^a^ Empirical 95 % confidence interval does not overlap with zero

## Discussion

 The results of this study, with Chinese nurses as participants, indicated that through the partial mediation of organizational commitment, psychological capital could affect job burnout. The finding that psychological capital can negatively affect job burnout is consistent with previous studies[12,16]. According to the Conservation of Resource Theory, job burnout results from either the lack of resources and inability to meet the job requirements or the imbalance between individual effort and payout[42]. Understandably, psychological capital, which is an important human resource, can prevent job burnout. As a composite concept, each dimension of psychological capital also correlates significantly with job burnout. For instance, Luthans et al. investigated Chinese employees and reported a positive correlation between the three positive mental states of hope, optimism, and resiliency, and work performance[15]. Evers, André Brouwers, and Tomic explored the relationship between self-efficacy and job burnout among teachers, and their results indicated that teacher self-efficacy was related to their burnout level, in which teachers with strong self-efficacy seemed to be more prepared to experiment and to implement new educational practices[43]. 

Optimistic individuals are less likely to experience burnout, as they make more attributions for positive events, are able to posit positive explanations for work events, possess positive attitudes, and are able to cope more easily in the face of different types of occupational stress[44,45]. Resilience is the ability of an individual to adjust positively to adversity, such that individuals with higher levels of resiliency recover more easily from frustration and failure, and this adaptive capability can significantly help one withstand the fatigue and emotional exhaustion caused by work stress[46]. Hope is the process of thinking about goals, the ways to achieve those goals, and the motivation to accomplish those goals. Research suggests that individuals with more hope typically have clear work objectives, formulate practical action plans to meet their objectives, and work hard to meet these objectives; hence, they are less likely to experience the negative effects of burnout[47,48]. Since psychological capital is the generic concept of self-efficacy, optimism, resiliency, and hope, nurses with greater psychological capital would naturally experience lower levels of burnout. This finding suggests the importance of nurturing self-confidence and positive coping styles among nurses at work and training them to handle stressful events. When nurses are hopeful about their jobs, have optimistic attitudes, and possess high levels of endurance and adaptability, their physical, emotional, and psychological depletion will be minimal, and they will not easily experience job burnout. 

The significant correlation between organizational commitment, on the one hand, and psychological capital and job burnout on the other, is consistent with the results of previous studies. In the current study, we focused on the moderating function of organizational commitment between psychological capital and job burnout. As attitude towards the organization, organizational commitment refers to the ground for the willingness of an individual to remain in the organization. Employees with greater levels of psychological capital are more likely to be dedicated to their assignments, to have a strong sense of duty, and to respond resolutely to adversity. They also identify more strongly with the team, enjoy more harmonious interpersonal relationships, and are more willing to contribute to the organization. Thus, such employees possess greater organizational commitment[26,27]. Wegge et al. found that a strong team identity is correlated with lower levels of emotional exhaustion and cynicism and higher levels of individual accomplishment[49]. We believe that individuals with greater psychological capital are more willing to contribute to the organization and to identify more strongly with the team. Therefore, they are able to receive all kinds of support from the organization and have higher levels of occupational happiness. The present study provides evidence in confirmation of this pathway of influence. 

Based on the results of the present study, we can conclude that enhancing psychological capital is an effective strategy for increasing organizational identity and reducing job burnout. As the psychological resource of an individual, psychological capital can indeed be developed and enhanced. Luthans et al. proposed a number of possible methods such development[12,13,15]. For example, an effective strategy for developing self-efficacy and self-confidence among employees is to allow employees to experience success. For example, management can help employees achieve goals or allow them to observe the successful results of continued efforts of others with similar backgrounds and circumstances. Another strategy is to train employees to use the stepping method to decompose personal goals into more manageable sub-goals, to enjoy the process of realizing the goal rather than to be solely preoccupied with the final result, and to be ready and willing to persevere when faced with obstacles and difficulties. Employees can also be trained to tolerate past events and accept their past mistakes, failures, and setbacks. Employees should be encouraged to appreciate the present and to be grateful for the positive aspects of their present lives. They should seek opportunities for future improvement and development, view future uncertainties as opportunities for development and progress, and face tomorrow with a positive, welcoming, and confident attitude. 

The present study has some limitations. We employed a cross-sectional rather than longitudinal design, and we do not have evidence for the dynamic influence of psychological capital on organizational commitment and job burnout. As China is a collectivist nation, organizational factors have a strong impact on employee occupational happiness. Further, whether the mediating function of organizational commitment between psychological capital and job burnout holds cross-culturally is a question well worth exploring in future studies.
